# Effects of Mesenchymal Stem Cell Transplantation on Cerebrospinal Fluid Biomarkers in Progressive Multiple Sclerosis

**DOI:** 10.1093/stcltm/szab017

**Published:** 2022-02-15

**Authors:** Panayiota Petrou, Ibrahim Kassis, Ariel Ginzberg, Michelle Hallimi, Dimitrios Karussis

**Affiliations:** Multiple Sclerosis Center/Neuroimmunology Unit, Department of Neurology, The Agnes-Ginges Center for Neurogenetics, Hadassah University Hospital, Jerusalem, Israel

**Keywords:** multiple sclerosis, mesenchymal stem cells, stem cells, neurofilaments (NF-L), neurodegeneration, neuroprotection

## Abstract

**Background:**

Neurofilament light chains (NF-L) were shown to serve as a reliable biomarker of neurodegeneration in multiple sclerosis (MS). The chemokine receptor CXCL13 was shown to correlate with CNS inflammatory activity and to predict the future progression of MS.

**Objective:**

To evaluate the levels of NF-L and CXCL13 in the cerebrospinal fluid (CSF) following treatment with mesenchymal stem cells (MSC) in patients with progressive MS.

**Methods:**

The CSF samples were obtained from 48 patients with progressive MS who participated in a double-blind randomized phase II clinical trial that tested the effects of intrathecal (IT) or intravenous (IV) transplantation of mesenchymal stem cells (MSC), at baseline (before the first injection of the MSC) and at 6 months following treatment with MSC, or sham treatment. The CSF specimens were tested in a blinded way, using a single-molecule array (SIMOA) technique.

**Findings:**

The CSF levels of NF-L were significantly lower at 6 months following treatment with MSC-IT when compared with the baseline, pre-treatment measurements (*P* = .026, Wilcoxon paired test). Nine out of 15 tested patients in the MSC-IT group had a reduction in NF-L levels of more than 50% (median decrease: −4449 pg/mL) when compared with 5/15 in the MSC-IV group (median decrease: −151 pg/mL) and 1/15 in the placebo group (median increase: +2450 pg/mL) (*P* = .001 for MSC-IT vs. placebo, chi-square test). CXCL13 levels were also reduced at 6 months following MSC-IT treatment but not to a statistically significant level.

**Conclusions:**

Our findings indicate possible neuroprotective effects of MSC transplantation in patients with MS.

**Clinical trial registration:**

NCT02166021

**Significance Statement**

Neurofilament light chains (NF-L) and CXCL13 were shown to serve as reliable biomarkers of inflammatory activity and neurodegeneration in multiple sclerosis (MS). The authors evaluated the levels of NF-L and CXCL13 in the CSF, following treatment with mesenchymal stem cells (MSC) in 48 patients with progressive MS. CSF levels of NF-L were significantly reduced at 6 months after a single intrathecal injection of MSC (median decrease: −4449 pg/mL, *P* = .026, Wilcoxon paired test). CXCL-13 levels were also reduced but not to a statistically significant level. These findings indicate possible in vivo neurotrophic effects of MSC in MS.

## Introduction

Following axonal damage in the central nervous system (CNS), neurofilament proteins (NF) released into the cerebrospinal fluid (CSF) provide a reliable indication of axonal damage and neuronal death. The most extensively NF subtype studied in this context is the neurofilament light chains (NF-L).^1^ Since NF are central components of the cytoskeleton of neurons, any neurological disease that leads to neuronal or axonal damage may cause an increase in the CSF levels of these proteins.

Indeed, high levels of NF-L were consistently found in the CSF of patients with multiple sclerosis (MS),^2^ suggesting that NF-L could be used as a biomarker of MS disease activity (including subclinical activity) and of the response to various MS treatments.^2-6^ Moreover, high serum NF-L levels in early MS were shown to predict a future increase in MS lesions and brain atrophy.^3,5^

The CXCR5 ligand CXCL13, the most potent B-cell chemoattractant, is detected in active MS lesions and in the CSF of patients with MS.^7^ CXCL13 was shown to be associated with disease exacerbations and unfavorable prognosis in MS and high CXCL13 levels predicted the conversion of clinical isolated syndrome (CIS) to MS.^7^

The use of stem cells in neurodegenerative diseases and particularly MS has been proposed during the last decade as a potential means for induction of neuroprotection and neuro-regeneration.^8,9^ Mesenchymal stem cells (MSC) have been the most used adult stem cells in MS clinical trials. Several small open or randomized studies^10-12^ provided indications of beneficial effects, including improvements in neurological functions. However, as for now, there is still no convincing evidence of objective neurotrophic and neuroprotective effects, induced by MSC, in vivo.

We report here the effects of MSC transplantation on the levels of NF-L and CXCL13 in the CSF of patients with active progressive MS.

## Methods

The CSF samples were obtained from 48 patients with progressive MS who participated in a double-blind randomized phase II clinical trial^12^ that tested the effects of intrathecal (IT) or intravenous (IV) transplantation of mesenchymal stem cells (MSC), at baseline (before the first injection of the MSC) and at 6 months following treatment with MSC, or sham treatment. The detailed treatment protocol of this trial is described elsewhere^12^ (details of MSC preparation and treatment protocol are presented in the Supplementary material). The samples of 4 patients were not technically testable for NF-L and the samples of 10, non-testable for CXCL13. The numbers of patients tested for NF-L in each group were 15, 15, and 14 for the MSC-IT, MSC-IV, and placebo groups, respectively, and 11, 14, and 13, respectively, for CXCL13. The CSF specimens were kept frozen at −20°C and then (after defrosting) tested in a blinded way, using a single-molecule array (SIMOA) technique and an HD-X Analyzer (QUANTERIX) for the evaluation of the concentrations of NF-L and CXCL13. This technique is widely accepted as the most sensitive method to detect these biomarkers (LLOQ = 0.316 pg/mL, pooled CV19%, mean recovery 102%, LOD = 0.5552 pg/mL, range 0.0152-0.108 pg/mL) and has been used in most of the previously mentioned studies in MS.^13^

## Findings

**Figure 1.**
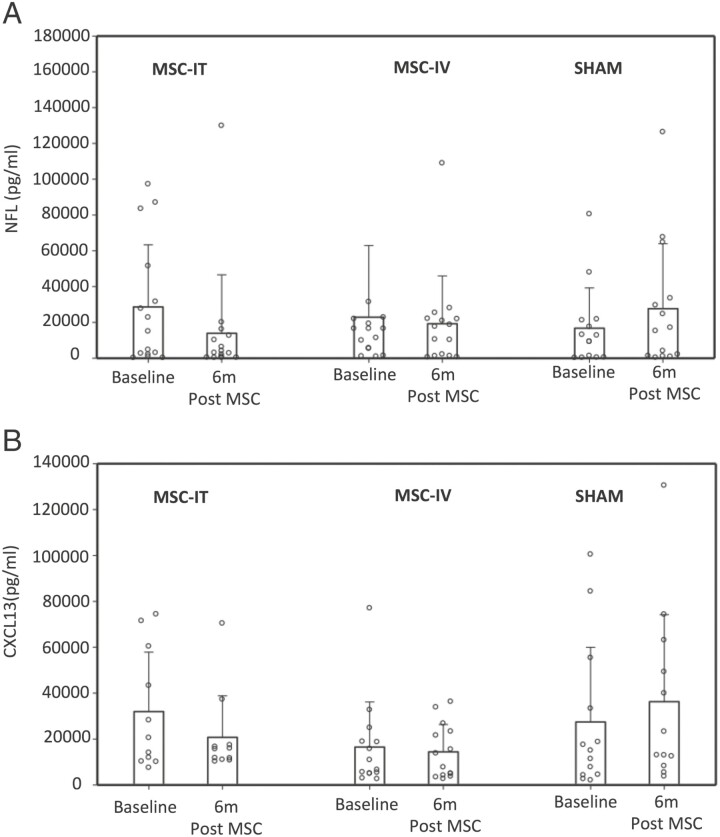
CSF levels of NF-L (A) and CXCL13 (B) at baseline and 6 months following intrathecal (MSC-IT) or intravenous (MSC-IV) treatment with MSC. Abbreviations: CSF, cerebrospinal fluid; MSC, mesenchymal stem cells; NF-L, neurofilament light chains.

The levels of NF-L (and CXCL13) did not differ significantly between the 3 groups at baseline (median: 14 937 pg/mL, 9845 pg/mL, and 10 920 pg/mL in the MSC-IT, MSC-IV, and placebo groups, respectively) (Table 1). CSF concentrations of NF-L were exceptionally high in almost all patients (>10 000 pg/mL in 8 patients in the MSC-IT group and in 7 in the MSC-IV and the placebo groups), indicating an active and aggressive disease, as also indicated by the deterioration in EDSS and MRI activity prior to the treatment.^12^ The levels of NF-L were significantly reduced at 6 months following treatment with MSC-IT when compared with the baseline pre-treatment measurements (*P* = .026, Wilcoxon paired test). The mean reduction was −14 723 pg/mL in the MSC-IT-treated group, −3801 pg/mL in the MSC-IV group (*P* = .955), and +10 905 pg/mL (increase) in the placebo group (*P* = .048) (Table 1; Fig. 1). Nine out of 15 tested patients in the MSC-IT group had a reduction in NF-L levels of more than 50% (median decrease: −4449 pg/mL) versus 1/15 in the placebo group (*P* = .001, chi-square test) (Table 1; Fig. 1A). Eight out of those 9 patients treated with MSC-IT with a >50% reduction in the NF-L levels were stable or improved in EDSS, and 7 out of 9 improved in the sum score of all the functional systems (FS) of the expanded disability status scale (EDSS), at 6 months; 8 out of 9 had an improved score even at 12-month follow-up (end of the study). However, no definite statistical correlation between clinical parameters and NF-L levels, could be detected, probably due to the small number of patients in each group.

**Table 1.** Changes in the concentrations of NF-L and CXCL13 in the CSF of each patient (pg/mL), 6-month post-MSC transplantation versus baseline.

Treatment group	Patient number	NF-L Baseline	NF-L 6 months	Delta change	CXCL13 Baseline	CXCL13 6 months	Delta change
MSC-IT	3	83 441	16 101	**−67** **340**	NA	NA	
	11	27 620	10 093	**−17** **527**	NA	NA	
	20	86 942	129 751	**42** **808**	20.5	10.23	**−10.27**
	23	22 714	12 515	**−10** **199**	10.19	15.59	**5.4**
	31	51 458	6219	**−45** **239**	74.31	11.58	**−62.73**
	38	14 937	3000	**−11** **936**	11.86	16.54	**4.68**
	40	859	4000	**3141**	10.06	15.89	**5.83**
	45	2000	946	**−1054**	28.21	17.31	**−10.9**
	4	31 527	2005	**−29** **522**	71.43	10.70	**−60.73**
	10	97 158	20 039	**−77** **118**	43.23	37.23	**−6**
	14	NA	NA		60.31	70.36	**10.05**
	21	188	263	**75**	13.98	11.69	**−2.29**
	26	178	220	**42**	NA	NA	
	36	4650	200	**−4449**	NA	NA	
	37	2591	350	**−2240**	NA	NA	
	47	3000	2705	**−295**	7.46	10.97	**3.51**
Mean		**28** **618**	**13** **894**	**−14** **723**	**28.01**	**21.74**	**−11.22**
Median		**14** **937**	**3000**	**−4449**	**17.24**	**15.74**	**−2.29**
MSV-IV	7	163 813	21 782	**−142** **030**	676.97	26.76	**−650.21**
	8	9844	108 845	**99** **000**	18.67	36.24	**0.35**
	17	22 683	21 999	**−683**	NA	NA	
	22	5240	27 934	**22** **693**	2.92	13.73	**10.81**
	29	16 465	20 872	**4407**	NA	NA	
	33	805	1283	**478**	6.55	7.68	**1.13**
	34	969	1203	**234**	5.53	4.90	**−0.63**
	46	NA	NA		32.65	33.81	**1.16**
	1	5573	2059	**−3514**	5.32	3.37	**−1.95**
	15	11 245	10 210	**−1035**	4.87	4.10	**−0.77**
	106	21 895	25 344	**3448**	18.82	21.53	**2.71**
	24	31 352	10 413	**−20** **939**	4.99	4.88	**−0.11**
	30	19 160	1177	**−17** **983**	10.86	2.59	**−8.27**
	35	836	532	**−303**	24.85	23.26	**−1.59**
	41	1000	1103	**102**	15.69	15.42	**−0.27**
	42	1343	450	**−893**	2.56	3.63	**1.07**
Mean		**20** **815**	**17** **014**	**−3563**	**59.37**	**14.42**	**−40.41**
Median		**9844**	**10** **210**	**−151**	**8.70**	**10.70**	**−0.06**
Placebo	5	NA	NA	NA	NA	NA	
	9	213	2087	**1874**	100.39	1088.32	**987.93**
	13	13 043	15 184	**2141**	7.74	23.24	**15.5**
	19	80 441	67 544	**−12** **897**	2.02	8.23	**6.21**
	25	411	300	**−110**	14.97	12.95	**−2.02**
	39	1240	4000	**2760**	4.42	12.45	**8.03**
	44	370	1082	**711**	33.23	49.23	**16**
	48	150	818	**668**	2.56	3.71	**1.15**
	2	9154	1272	**−7882**	11.30	63.03	**51.73**
	6	12 684	16 993	**4309**	18.71	39.88	**21.17**
	12	17 458	24 610	**7152**	84.32	130.43	**46.11**
	18	21 159	126 291	**105** **132**	17.47	12.89	**−4.58**
	27	21 694	33 400	**11** **705**	4.21	5.37	**1.16**
	28	47 903	64 649	**16** **746**	55.34	74.23	**18.89**
	32	9145	29 503	**20** **358**	NA	NA	
	43	NA	NA		NA	NA	
Mean		**16** **790.73**	**27** **695.80**	**10** **905**	**30.26**	**44.55**	**89.79**
Median		**10 919.60**	**16** **088.89**	**2450**	**17.47**	**24.40**	**15.50**

The differences after the treatment are shown in bold to be clearer to the reader.

Abbreviations: CSF, cerebrospinal fluid; MSC, mesenchymal stem cells; NA, not applicable; NF-L, neurofilament light chains.

CXCL13 levels were reduced at 6 months following MSC-IT treatment (mean change: −11.22 pg/mL, median: −2.29) and significantly increased in the placebo group (median change: +89.8, median: +15.5, *P* = .012, Wilcoxon signed rank test). However, this trend of reduction of CXCL13 in the MSC-IT group did not reach statistical significance compared with the change (increase) observed in the placebo group. No changes in the CXCL13 levels were detected in the MSC-IV treatment group (*P* = .975) (Table 1; Fig. 1B).

## Discussion

The changes in the NF-L levels in the CSF following IT injection of autologous MSC, are—to our knowledge—novel and firstly described in the literature. The only report of in vivo effects of MSC on the levels of neurofilaments derived from a study where the stem cells were injected intravenously; similarly to our data, the authors of this trial did not detect significant changes in the serum levels of NF-L, following MSC-IV treatment.^14^

Our findings, taken together with the neurological benefits observed in other parameters of MS progression (brain volume changes in magnetic resonance imaging [MRI], improvements in cognition, disability, and in the functional MRI),^12^ seem to provide an additional hint of possible neuroprotective or neurotrophic effects, induced by MSC.

Several immunomodulating treatments of MS were shown to induce moderate reductions in the serum levels of NF-L.^6^ It has been hypothesized that this is probably related to the suppression of inflammation and of new relapses, which results in the reduction of neuronal damage. In our study, the moderate (and not significant) changes in the CXCL13 levels that were induced by MSC treatment (Table 1; Fig. 1A) do not support the possibility of immunomodulation as the prominent mechanism involved in the reduction of NF-L levels. However, since MSC were shown to induce strong immunomodulatory effects in vitro^15^ and in vivo,^12^ such possibility cannot be excluded and should be further evaluated by testing additional immune parameters. Nevertheless, the magnitude of the effect of MSC-IT treatment on the NF-L levels in the CSF indicates amelioration of neurodegeneration, regardless of whether this is related to anti-inflammatory or neurotrophic/neuroprotective mechanisms.

The IT injection of MSC brings the stem cells in closer proximity to the inflammatory areas of the CNS when compared with the IV way of administration. This could explain the observed superiority of the MSC-IT over MSC-IV treatment on the suppression of the NF-L levels in the CSF (and on several other parameters of MS activity, described in our clinical trial^12^) and may be related to either downregulation of the compartmentalized CNS inflammation (which has been shown to be one of the main mechanisms that drive neurodegeneration and accumulation of disability in progressive MS^16^) or direct neurotrophic/neuroprotective effects of the MSC on the CNS.

The main strength of our study is that it represents the first objective in vivo documentation of neuroprotective/neurotrophic effects, induced by MSC in patients with MS, demonstrated with the use of a CSF biomarker. A possible limitation of our findings it could be that the reductions in the tested biomarkers may be theoretically attributed to a “regression to the mean” or a stabilization of MS following the significant activity and progression that was observed in the year prior to inclusion. However, the fact that the reductions of NF-L were only seen in the MSC-IT group, whereas in the placebo-treated patients, the NF-L levels increased (indicating continuation of neurodegeneration), does not support such possibility. The proportions of patients with MS progression or activity during the year prior to their inclusion were identical in the 3 groups of the trial and their previous treatments’ repertoire, similar^12^, excluding therefore the possibility that the observed changes in NF-L levels could be attributed to differences between the study groups in these parameters. An additional, obvious limitation of our study is its rather small size and short duration. Larger-scale studies with a longer follow-up period are needed to confirm these observations.

## Supplementary Material

szab017_suppl_Supplementary_Material

## Data Availability

The data underlying this article will be shared on reasonable request to the corresponding author.
