# Supplementary material for: Effects of Mesenchymal Stem Cell Transplantation on Cerebrospinal Fluid Biomarkers in Progressive Multiple Sclerosis
Source: Stem Cells Transl Med. 2022 Feb 15;11(1):55–8. doi: 10.1093/stcltm/szab017 (PMC8895488; doi:10.1093/stcltm/szab017)
Supplement: szab017_suppl_Supplementary_Material [file szab017_suppl_supplementary_material.docx]

**Supplementary Material**

Preparation of MSC and treatment protocol

Fresh bone marrow (BM) was aspirated according to the routine medical center procedure from the patient’s iliac crest under local anesthesia and sedation, following testing negativity for HBV, HCV, and HIV. BM (~100 mL) was aspirated using aspiration needles into heparin-containing sterile bags (Macopharma, USA). The aspirated BM was transferred immediately to the GMP facility and labeled by the physician or by the attending technical assistant. BM aspirates were transferred from the heparin-containing BM aspiration bags into sterile 50 mL conical tubes (Corning, USA) using 2 spike tubing sets (Macopharma, USA) and diluted 1:1 (v:v) in Hanks’ balanced saline solution (HBSS, Sigma-Aldrich), and mononuclear cells (MNCs) were separated from the total BM inoculum by Ficoll density gradient (1.073 g/mL) centrifugation (GE Healthcare, USA). Diluted BM was transferred to barrier-containing 50 mL tubes (LEUCOSEP, Greiner Bio-One, Germany) prefilled with 15 mL of Ficoll and centrifuged at 1000*g* for 10 minutes at 24°C. The MNC layer was removed using sterile pasture pipette (Greiner Bio-One, Germany) and transferred to 50 mL sterile tubes and diluted with 30 mL CTS DPBS. Cells were centrifuged twice at 1000*g* for 10 minutes at 24°C and re-seeded into “complete culture media” containing NutriStem XF Basal Media (Biological Industries, Israel) supplemented with NutriStem Supplement media for further processing. MNCs were counted using hematocytometer, and cell viability was evaluated using trypan-blue dye staining (Sigma-Aldrich, Israel). MNCs were washed and re-suspended with NutriStem XF complete media and seeded on CellStack (636 cm^2^, Corning, USA) pre-coated with Attachment Solution XF. At this stage, non-adherent MNCs were floating in the culture supernatant, and plastic-adherent MSCs were attached to the flask surface. The culture supernatant containing the non-adherent MNCs was removed, and the adherent cells were gently washed with 100 mL DPBS. The step from MNC seeding to hMSC harvesting was designated as Passage 0 (P0). The P0 cells were incubated in a 37°C/5% CO_2_-humidified incubator, and the growth medium was replaced twice a week, with fresh complete NutriStem XF growth medium until the culture reached 80%-90% confluence but for no more than 12 days. Cells were subcultured at regular intervals when the culture reached 80%-90% confluence. Each subculture cycle was counted as a new passage. The cultures could be subcultivated further up to Passage 3. For sub-culturing MSC, the culture supernatant was removed from the flask and a CTS TrypLE Select solution was added to each flask. The flask was incubated for 8 minutes at 37°C, and a culture medium was then added to each CellStack to inactivate the enzymatic action. The detached cell suspension was transferred into centrifuge tubes, washed, re-suspended in the growth medium new CellStack. The cultures were then incubated in a 37°C, 5% CO_2_-humidified incubator for further culturing. A few days before cryopreservation, cells were characterized by FACS for human MSC markers as well as a biopotency test of mixed lymphocyte reaction (MLR) was performed. At the end of the process before cryopreservation, cells were tested for sterility, mycoplasma, and endotoxins. Cells were released for treatment upon receiving the results of the tests and according to the release criteria. Each cell batch was released with a certificate of analysis document (CoA).

The cultured cells were diluted with normal saline and transferred to 1 of the 2 syringes that were prepared for each patient (according to the treatment group assignment by the CRO). One syringe contained MSCs (1 × 10^6^/kg of body weight) re-suspended in 3 mL of normal saline and 1 syringe contained only normal saline. To ensure blinding, the treating physician received from the Laboratory Unit (according to the randomization number), 2 sealed syringes covered with black adhesive, for every patient, at each treatment cycle. The full 3 mL of the content in the sealed syringe were injected into the cerebrospinal fluid via lumbar puncture at the level of L4-5, using a 20-gauge needle and 3-way cannula. The 3 mL of the second syringe were injected into an aluminum-covered 500 mL sac with normal saline and infused into the patient for more than 30 minutes using a 20-gauge vein catheter. A volume of 3 mL of CSF was removed for biomarkers testing. During the 2 phases of the trial, each of the 48 patients received 1 intravenous and 1 intrathecal injection (of which, one or both was placebo), at each of the 2 treatment cycles.
